# Synovial Chondromatosis in a Young Athlete: A Report of a Rare Case

**DOI:** 10.7759/cureus.53173

**Published:** 2024-01-29

**Authors:** Vivek H Jadawala, Sanjay Deshpande, Salahuddin Ahmed, Sachin Goel, Anmol Suneja

**Affiliations:** 1 Department of Orthopaedic Surgery, Jawaharlal Nehru Medical College, Datta Meghe Institute of Higher Education and Research, Wardha, IND

**Keywords:** knee joint, synovial hypertrophy, joint pathology, osteochondromatosis, synovitis, arthroscopy, synovial chondromatosis

## Abstract

Synovial chondromatosis is a rare and benign disorder that involves the synovial lining of joints, synovial sheaths and bursae. The synovial layer of the joint is affected by a metaplastic process which in turn converts it into cartilagenous tissue. Eventually, it gets dislodged and transformed into a loose body in the large joints. We report the case of a 24-year-old young athlete who presented with complaints of painful movements and restriction of joint movements associated with a growing deformity in the right knee joint. This case report aims to describe a rare synovial pathology that necessitated arthroscopic synovectomy and diagnostic arthroscopy to treat, particularly in younger individuals. The atypical feature, in this case, was metaplastic development from the peripheral joint capsule attached to the surrounding cartilage, which, to the extent that the authors are aware, has only been documented in one instance in the record. Magnetic resonance imaging (MRI) was performed which demonstrated evidence of the joint effusion, synovial hypertrophy and a loose calcific body just anterior of the distal femoral condyle causing pressure over the patellar tendon anteriorly as well as a hyper-dense cyst in the popliteal region. Treatment often requires partial or complete synovectomy with either an arthroscopic or open approach.

## Introduction

Synovial chondromatosis, also called synovial osteochondromatosis, is a rare and benign disorder that involves the synovial lining of joints, synovial sheaths and bursae. It involves the synovium's metaplastic process that turns it into cartilage and causes it to get dislodged and transform into a loose body [1,2]. Synovial chondromatosis can arise in either joint in the body, but more commonly involves the knee, hip, elbow and shoulder joints in decreasing frequency [3]. Larger joints are commonly affected by primary synovial chondromatosis around the third to fifth decade of life, while small joints can also be affected and manifest earlier in life [4].

Patients commonly complain of pain that is mild to moderate in intensity, diffuse swelling around the involved joint and gross restriction of joint movements. Affected individuals are normally in their third to fifth decade of life [5]. However, there are cases where the involvement has also been reported in childhood [6,7]. The incidence in males is twice as frequent as in females [8]. Unilateral joint involvement is most common; however, bilateral occurrences are also reported [9-11]. The diagnosis suspected clinically is confirmed based on imaging modalities such as plain radiographs, ultrasound, computed tomography scans and MRI. The gold standard diagnostic method is MRI because of its superiority in enhancing soft tissue contrast [12].

This case report aims to describe a rare synovial pathology that necessitated arthroscopic synovectomy and diagnostic arthroscopy to treat, particularly in younger individuals. The atypical feature, in this case, was metaplastic development from the peripheral joint capsule attached to the surrounding cartilage, which, to the extent that the authors are aware, has only been documented in one instance in the record.

Synovial chondromatosis presents as the gradual, insidious onset of mono-articular joint discomfort and stiffness [5]. If the complaints are left untreated, they could lead to a reduction in movement range, effusions, crepitation, and eventually locking of the joint. Long-term osteoarthritis may lead to secondary synovial chondromatosis [2].

## Case presentation

A 24-year-old young athlete, who played kabaddi (a contact team sport played between two teams of seven players) in a district professional sports team, presented with complaints of painful movements and restriction of joint movements associated with growing deformity in the right knee joint. The patient describes a swelling that first appeared about two years ago and was associated with a fever. The onset of the patient’s symptoms was insidious and they progressed gradually in its severity. The patient received some treatments for the same from general practitioners, but his complaints were not alleviated. The patient also mentioned some surgical interventions done in another hospital about 11 months ago.

On clinical examination, the patient had a fixed deformity of 15 degrees in flexion with further flexion possible up to 80 degrees. The attitude of the right knee was in 15-20° flexion with apparent atrophy of quadriceps muscles. There was diffuse swelling of the knee with fullness in the supra-patellar and para-patellar region as well as popliteal fossa. There was a previous surgical scar of arthroscopic portals over the anteromedial and anterolateral aspects of the knee joint. There was no varus or valgus deformity.

**Figure 1:**
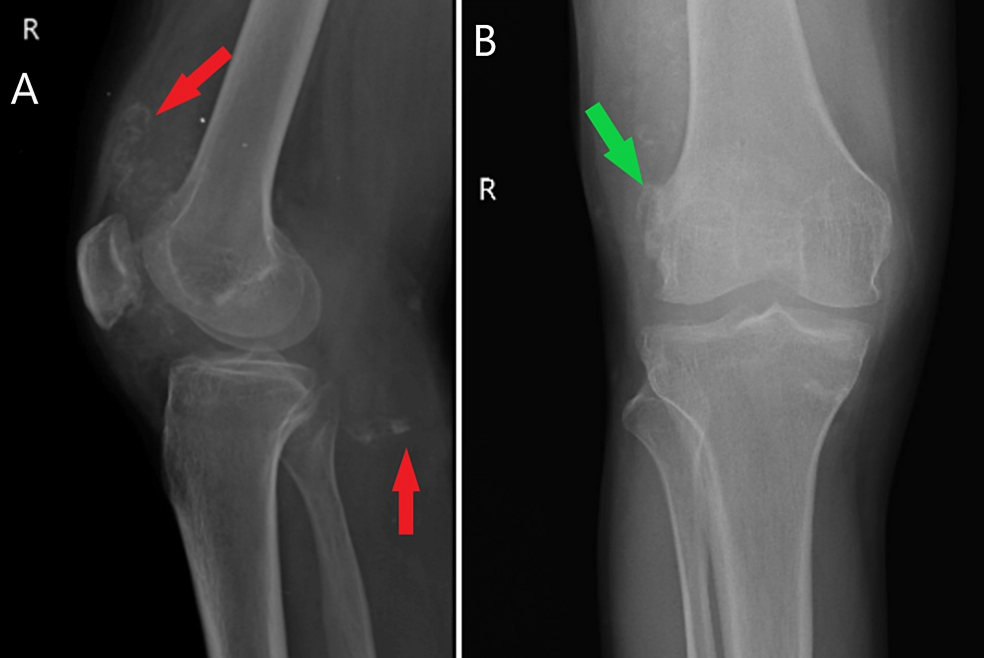
Plain radiograph (lateral and anteroposterior views) of the right knee. (A) and (B) showing radio-dense shadows anterior to the distal femoral condyle, proximal to the patella and in the popliteal fossa (red arrows). Osteophyte over lateral femoral condyle proximally (green arrow).

On palpation, there was no local rise in temperature. The tenderness was present along the medial and lateral joint line with probable joint effusion. There was multiple, somewhat movable soft-tissue swelling palpated just proximal to the patella, which was extending to the medial aspect of the joint and midline beneath the patellar tendon. Irregular soft tissue mass could be palpated along the edges of the lateral condyle of the femur. An irregularly thickened synovial membrane was palpated over the right knee joint. During flexion, mobile nodular masses were palpable over the proximal and medial to the joint line. The range of flexion was 15 degrees to 80 degrees. Instability tests to rule out ligament injury were performed and found to be negative. Distal neurovascular status was found to be intact. A plain radiograph of the right knee showed a broad radio-opaque shadow of a loose body in front of the distal femoral condyle proximal to the patella with irregularity of the articular margin of the distal femur as seen in Figure 1.

**Figure 2:**
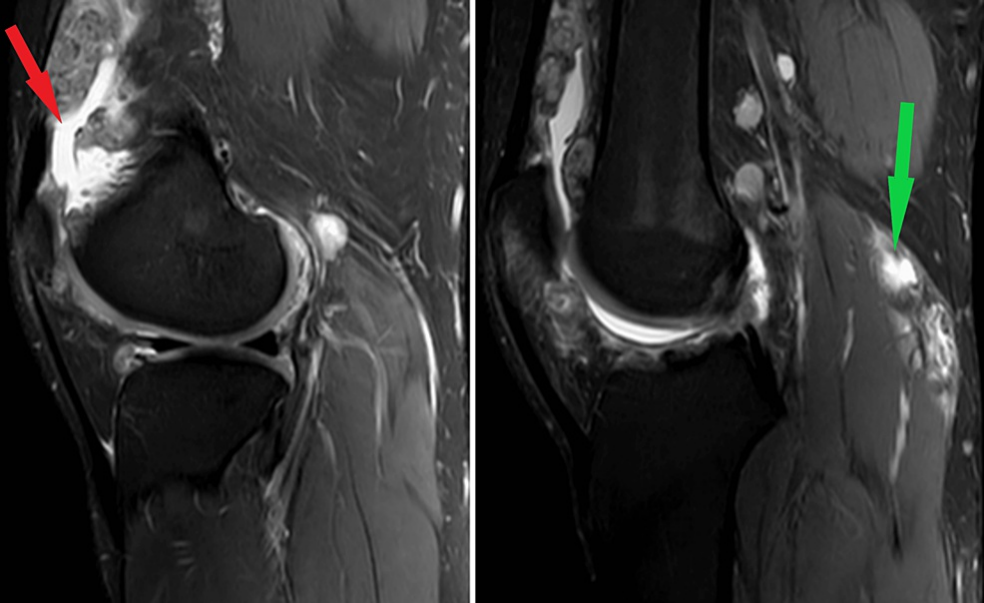
Sagittal sections of MRI of the right knee joint showing joint effusion (hyper-intense areas) with proliferative chondroid nodules of the synovium (appearing iso-to hypointense) and nodularity lining the synovium anterior to the distal femur condyle and the patello-femoral articular margin proximal to the patella in quadriceps tendon (red arrow). Joint effusion and popliteal cyst (hyper-intense cystic swelling) are visualized in the popliteal fossa (green arrow).

Radio-opaque shadows are seen in the supra-patellar pouch, infra-patellar region and popliteal fossa seen in Figure 2. Arthritic changes are seen in the radiographs.

**Figure 3:**
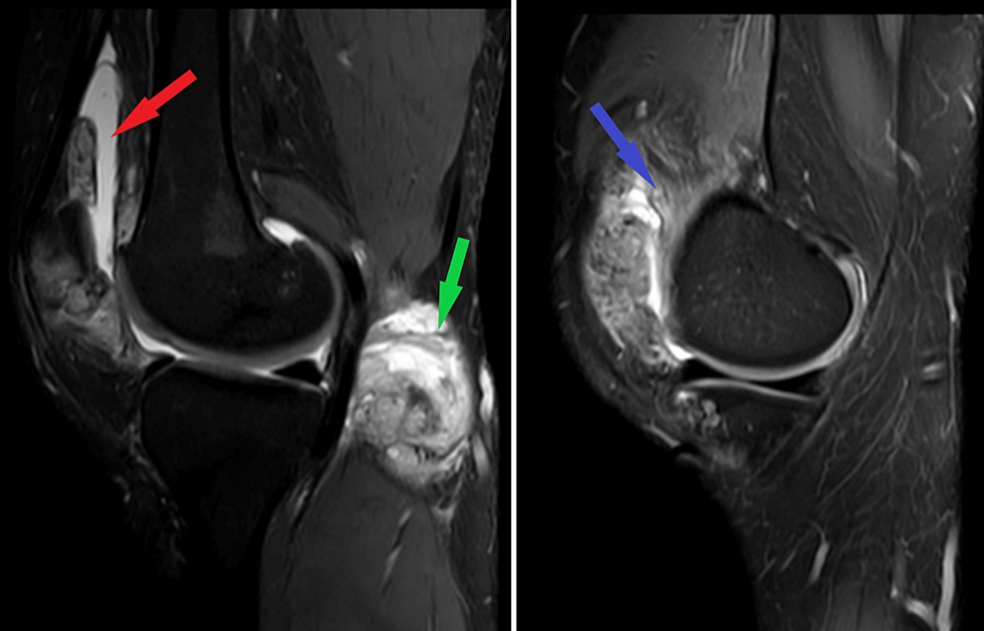
Sagittal section of MRI right knee joint showing popliteal cyst (hyper-intense cystic swelling) in the popliteal fossa (green arrow) as well as joint effusion (hyper-intense area) anterior to the distal femoral condyle (red arrow). The thickening of the synovial membrane with nodularity is also visualized (blue arrow).

MRI was performed which had evidence of the joint effusion, synovial hypertrophy and a loose calcific body just anterior of the distal femoral condyle causing pressure over the patellar tendon anteriorly as well as hyper-dense cyst in the popliteal region as shown in Figure 3.

**Figure 4:**
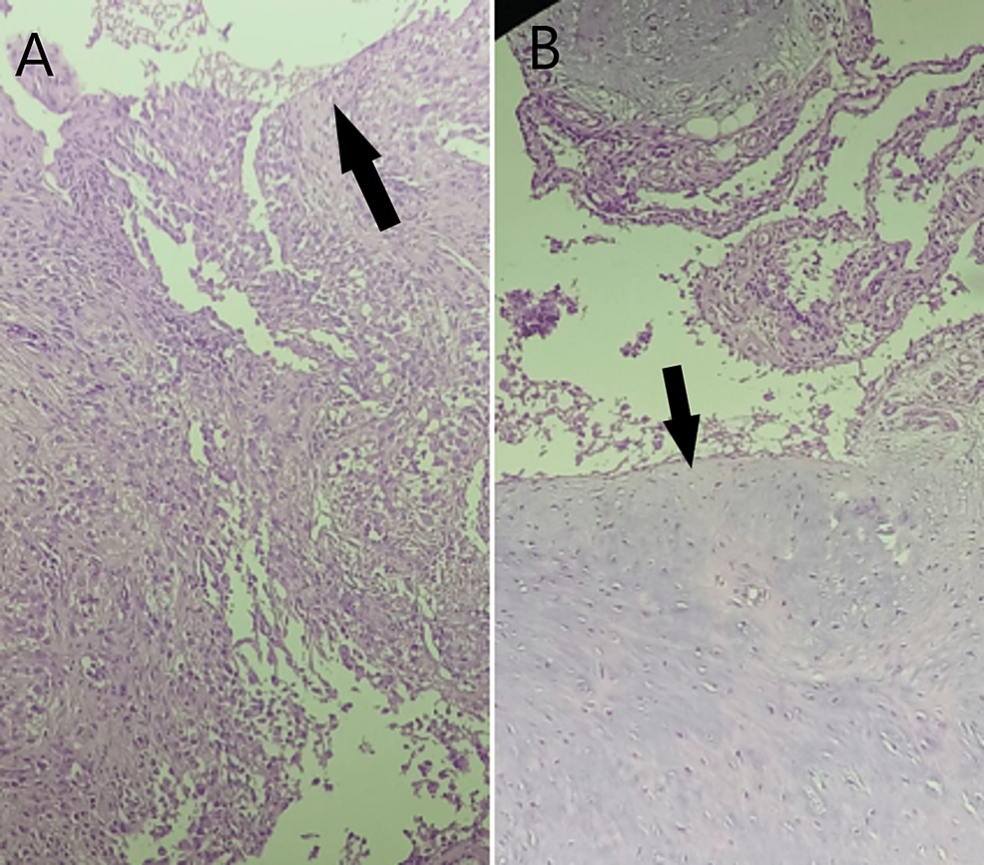
A & B - Histopathological section of synovial tissue from the periphery showing irregular and thickened cellular architecture (black arrow) (H&E stained, 40X). H&E: Hematoxylin and eosin

**Figure 5:**
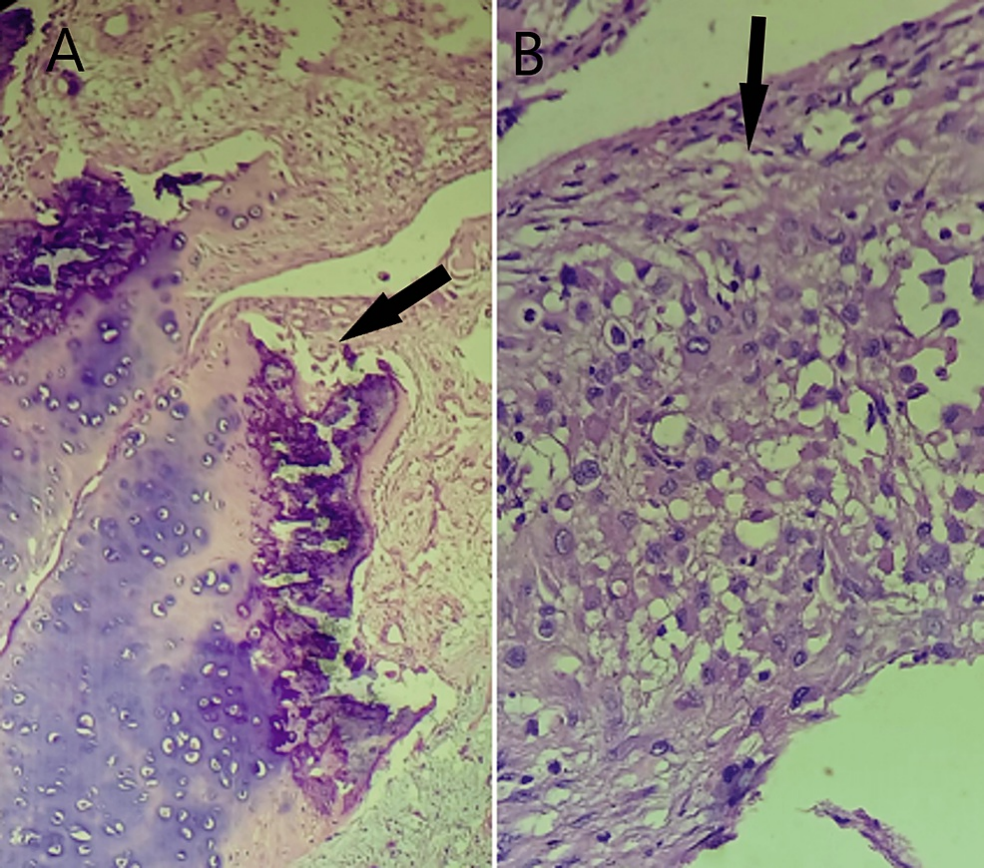
Histopathological section showing marginal tissues of synovial chondromatosis with hypertrophied synovium (black arrows) (H&E stained, 40X). H&E: Hematoxylin and eosin

A surgical plan was decided and arthroscopic debridement with synovectomy using anterior portals (antero-medial and antero-lateral) was considered. Arthroscopic debridement, removal of loose bodies and thickened synovium was done which was later sent for histopathological examination. Intra-operatively, there were diffuse calcifications present in the knee joint and suprapatellar, medial gutter and lateral gutter. Arthroscopic removal of calcified cysts was done, debridement was done, and the extensor tendon was freed of calcified cysts. A 2 cm x 3 cm big loose body was excised which was present underneath the patellar tendon and lateral retinaculum. Along the margins of the lateral condyle of the femur, there were irregular, thickened nodular synovial tissues present which were removed. Hypertrophied and unhealthy synovial tissue was removed from all the compartments. The loose bodies excised along with the synovial tissue taken out were sent for histopathological evaluation and synovial chondromatosis associated with papillary hyperplasia of the synovial tissue was confirmed as the final diagnosis as seen in Figure 4 and Figure 5.

Post-operatively, the patient was advised knee range of movement and quadriceps and hamstring strengthening exercises. The patient was followed up at the first, third and fifth months. At the third month follow-up, the patient’s active range of knee flexion-extension was 0-130° without any discomfort. There was no evidence of recurrence in the fifth month after the operation.

## Discussion

An uncommon benign, relatively reactive disorder known as synovial chondromatosis, is characterised by the development of cartilaginous structures inside the synovial membrane and subsynovial fibrous tissue of the major joints.

Synovial chondromatosis is characterized by three stages in the disease progression [2]. In stage 1, synovial metaplasia with active inflammation of the synovium and loose bodies are not formed yet. In stage 2, there is active inflammatory synovitis with the process of early formation of intra-articular or peri-articular loose bodies that are still made up of cartilage tissues. And in stage 3, the calcification of loose bodies occurs as well and there is a decrease in synovial inflammation.

In a joint as complex as the knee, the anatomic location as well as extension of the synovial involvement may also vary. The involvement may be extra-articular, intra-articular or both intra as well as extra-articular [5,13]. The condition's severity can range from only compromising the cruciate ligaments to severely damaging the whole knee joint [13-15]. Synovium of the affected joint may have nodular metaplastic growths adhered to it [5,6,9,11,12,16,17]. The case reported in the article, however, has a large number of nodular growths that originate from the synovial membrane at the edge of the joint and are attached to the joint line and nearby articular cartilage. Allard et al. [18] performed an immune-histochemical study that showed that a wedge-shaped vascular tongue of tissue was covering the cartilage surface in all joints at the junction with the synovial membrane. This bordering cartilage tissue continued into the neighbouring synovium and shared immunohistochemistry characteristics with it [18].

Treatment of synovial chondromatosis is often surgical. Both minimally invasive arthroscopic as well as open surgical methods with partial or subtotal synovectomy are utilized for management [3,16]. The comparative results of synovectomy along with loose body removal have shown better results as compared with loose body removal alone [16]. The presence of osteoarthritis along with synovial chondromatosis demands total knee arthroplasty depending on the severity of joint involvement [5].

The differential diagnosis includes teno-synovial giant cell tumour, synovial chondrosarcoma, siderotic synovitis, tumoral calcinosis, peri-articular melorheostosis, synovial hemangioma, pigmented villonodular synovitis, and lipoma arborescens [19]. Appropriate clinical, radiological and histopathological diagnosis helps in accurately differentiating the cases of synovial chondromatosis.

Although rare, synovial chondromatosis may be complicated and can lead to malignant transformation (chondrosarcoma) of the synovium and secondary osteoarthritis of the joint. Recurrence has also been observed in the vast majority of cases [16,20].

## Conclusions

Synovial chondromatosis is a rare and benign condition and its occurrence in young adults in the second decade of life is rarely reported. An atypical feature of the synovial membrane with metaplastic growth is not very commonly seen. Treatment often requires partial or complete synovectomy with either an arthroscopic or open approach.
